# Supplementary material for: The impact of angles of insonation on left and right ventricular global longitudinal strain estimation in fetal speckle tracking echocardiography
Source: PLoS One. 2023 Jul 12;18(7):e0287003. doi: 10.1371/journal.pone.0287003 (PMC10337891; doi:10.1371/journal.pone.0287003)
Supplement: S1 Table — (DOCX) [file pone.0287003.s001.docx]

**Supplementary table 1**. Median frame rates and mean global longitudinal strain for the sensitivity analysis.

|  | **Angle of Insonation** | | |
| --- | --- | --- | --- |
|  | **Oblique** | **Perpendicular** | **Up/down** |
| Median # frames / second (IQR)  Median # frames / cardiac cycle (IQR) | *n=43* | *n=11* | *n=20* |
|  | 122 (111-139) | 119 (84-125) | 95 (88-126) |
|  | 50 (44-55) | 50 (32-54) | 39 (36-52) |
| Mean GLS left ventricle (95%CI)  Mean GLS right ventricle (95%CI) | -22.94 (-26.10 ̶ -19.81) | -30.61(37.46 ̶ -23.75) | -20.79 (-23.81 ̶ -17.78) |
|  | -20.47 (-23.68 ̶ -17.26) | -21.82 (-29.68 ̶ -13.95) | -18.88 (-22.86 ̶ -14.90) |

IQR = interquartile ranges, 95%CI = 95% confidence interval.
